# Social support of adults and elderly with chronic kidney disease on dialysis

**DOI:** 10.1590/1518-8345.0411.2752

**Published:** 2016-08-08

**Authors:** Simone Márcia da Silva, Natalia Fernanda Braido, Ana Carolina Ottaviani, Gabriela Dutra Gesualdo, Marisa Silvana Zazzetta, Fabiana de Souza Orlandi

**Affiliations:** 1 Master's Student, Faculdade de Medicina de Ribeirão Preto, Universidade de São Paulo, Ribeirão Preto, SP, Brazil.; 2 Undegraduate student in Gerontology, Universidade Federal de São Carlos, São Carlos, SC, Brazil.; 3 Master's Student, Departamento de Enfermagem, Universidade Federal de São Carlos, São Carlos, SP, Brazil.; 4 PhD, Adjunct Professor, Departamento de Gerontologia, Universidade Federal de São Carlos, São Carlos, SP, Brazil.

**Keywords:** Social Support, Renal Insufficiency, Chronic, Health of the Elderly, Adult Health

## Abstract

**Objective:**

to evaluate the instrumental and emotional social support of patients with chronic kidney disease on hemodialysis.

**Method:**

descriptive cross-sectional study. The sample was sized for convenience and included 103 participants under treatment in a Renal Replacement Therapy Unit. Data were collected through individual interviews, using the Social Support Scale.

**Results:**

the mean scores of the emotional and instrumental social support were 3.92 (± 0.78) and 3.81 (± 0.69) respectively, an indication of good support received. The most frequent sources of instrumental and emotional social support mentioned by participants were partners, spouse, companion or boyfriend and friends.

**Conclusion:**

patients with chronic kidney disease have high social support, both instrumental and emotional, and the main support comes from the family.

## Introduction

Chronic Kidney Disease (CKD) is defined as a progressive and irreversible loss of kidney function, which is classified according to the glomerular filtration rate^1^. CKD is considered a public health problem worldwide^1^ ^-^ ^2^. According to the latest census released by the Brazilian Society of Nephrology, it is estimated that in 2011, approximately 91,300 patients were on dialysis in Brazil, and the estimated number of patients who started treatment in 2010 was 18,972. Regarding the dialysis type, about 90% of the patients investigated in the 2010 Census were on hemodialysis in the various units that responded to the questionnaire^3^.

Despite major technological advances in the treatment of CKD and increased survival of these patients, none of the existing methods is curative. In other words, deal with the chronicity of the disease and the limitations imposed by the treatment is necessary for the patients on replacement therapy and has an important psychological impact for these individuals. Hemodialysis, in particular, is responsible for a restricted daily routine since it imposes limitations on individuals affecting the biological, psychological and social aspects of their lives. This leads to a break in their lifestyle, causing the need to adapt to this new condition^4^.

Another aspect that seems to influence the outcome and quality of life of patients on hemodialysis is the perceived social support level. The first category refers to the availability of assistance from other people in the management or resolution of practical or operational situations of everyday life, such as the material and financial support or help with several day-to-day activities^6^.

In the literature, two categories of social support have prevailed: instrumental and/or emotional. The first category refers to the availability of the assistance of the others in the management or resolution of practical or operational situations of everyday life, such as the material and financial support or help with several day-to-day activities. The emotional support consists of behaviors such as listening, paying attention or keep company, which makes the person feel cared for and/or estimated^7^. The social support network is a web of social relationships that each individual keeps, including the closest people, such as family and close friends^8^.

In view of the theoretical framework presented and the relative earliness of research, this study aims to produce knowledge on the social support of patients with chronic kidney disease on hemodialysis. The survey question that this study intends to answer is: What is the of social, instrumental and emotional support level of the patients with chronic kidney disease on hemodialysis?

In search of publications on the assessment of social support of patients with chronic kidney disease, using quantitative research tools, it was found only one study on renal transplant patients. There has been a lack of studies related to social support of patients with chronic kidney disease^9^. 

It is believed that the assessment of social support of patients with chronic kidney disease on hemodialysis may serve to ensure that health professionals, especially nurses, social workers and psychologists, can improve the clinical follow up of these individuals with an efficient treatment adherence, leading to considerable clinical results and therefore a better quality of life. Importantly, there are no quantitative studies aiming to evaluate the emotional and instrumental social support of patients with chronic kidney disease on hemodialysis. 

Based on the above, the aim of this study was to characterize patients with chronic kidney disease, in terms of sociodemographic and health characteristics, and assess the instrumental and emotional social support provided of patients with chronic renal failure on hemodialysis.

## Method

This is a descriptive, cross-sectional study, developed in a Renal Replacement Therapy Unit, in a municipality in the State of São Paulo. The Nephrology Service serves 150 patients beneficiaries of the Unified Health System and people enrolled in private health insurances, covering the cities of São Carlos, Porto Ferreira, Descalvado, Ibaté, Ribeirão Bonito and Itirapina. 

The convenience sample consisted of 103 participants, in total, who met the following criteria: be 18 years or older, be diagnosed with terminal CKD and be on hemodialysis. After the invitation, the aim of this study was informed and the possible questions were answered. After they agreed to participate in the study, all respondents signed the informed consent form.

Data collection was conducted using a Questionnaire of the Sociodemographic and Clinical Characterization and the Social Support Scale for People Living with the Human Immunodeficiency Virus (HIV) - adapted for renal patients. The Questionnaire for the Sociodemographic and Clinical Characterization consists of questions regarding the identification (name, age, gender), demographic data (or self-declared, schooling, family income and religion) and clinical data (baseline diseases and willingness to undergo a kidney transplantation).

The Social Support Scale for People living with the Human Immunodeficiency Virus (HIV) was developed in Canada^10^, validated for the Brazilian context, in 2006^11^, and adapted for this study. It is worth mentioning that the only adjustment made in the instrument was on the instruction sheet, replacing the term "HIV positive" by "chronic kidney disease". A similar process was performed in a study of patients with heart failure^12^.

 The scale assesses the perception of people on social support received and has 22 items or specific questions, 10 items related to the instrumental support and 12 items related to emotional support^13^. The answers are based on a Likert-type scale with 5 choices both for the availability of support (1 = never, 2 = rarely, 3 = sometimes, 4 = often, 5 = always) and for the satisfaction of support (1 = very dissatisfied, 2 = dissatisfied, 3 = neither satisfied/nor dissatisfied, 4 = satisfied, 5 = very satisfied). The instrument contains, in the end, an open question in order to identify other types of social support received by individuals, not listed yet^10^ ^-^ ^14^.

For composition of the scale, the scores are calculated by the arithmetic mean of the values of the items corresponding to each factor (availability and satisfaction), so that these range from 1 to 5 for the emotional support and for the instrumental support. It was established that the higher the value, the greater the perceived availability and satisfaction with the support evaluated^15^.

These instruments have been applied before the hemodialysis session, or, if not possible, in the first two hours of treatment. Given the possibility that some of the participants had visual problems and/or low level of education, the application of the instrument was conducted through individual interviews, in the period from June to August 2012.

Data were entered into an Excel spreadsheet and analyzed using the Statistical Package for the Social Sciences software (SPSS for Windows) version 19.0. For descriptive data analysis, position measurements (average, minimum and maximum) and dispersion (Standard Deviation) were calculated. Cronbach's alpha (α) was used to check the internal consistency of the Social Support Scale for People Living with the Human Immunodeficiency Virus (HIV).

The Research Ethics Committee of the Federal University of São Carlos approved this study (Opinion nº 42.155/2012). The development of this study met the standards of ethics on human research.

## Results

The study included 103 participants with an average age of 54.81 years, with a predominance of males (67.0%) and most patients had 1 to 8 years of study (54.4%). As for the monthly income, most (65.6%) received only a minimum wage (minimum wage in the period of data collection: R$ 724.00 per month). Catholicism was the predominant religion, n=64 (62.1%), and 59.2% were practicing Catholics (Table 1).

**Table 1:** Description of the sociodemographic characteristics of the patients with chronic kidney disease. São Carlos, SP, Brazil, 2015.

Variable	Distribution by category	N	%
Age	24-33 years	09	8.7
	34-43 years	12	11.5
	44-53 years	23	22.2
	54-63 years	32	31.1
	64-73 years	18	17.8
	74-85 years	09	8.7
Sex	Male	69	67.0
	Female	34	33.0
Schooling	Illiterate	10	9.7
	1 to 8 years	56	54.4
	9 to 11 years	26	25.2
	12 or more	11	10.7
Self-declared ethnicity	White	72	69.9
	Black	22	21.4
	Brown	09	8.7
Family Income (MW)* n=87†	Up to 1	57	65.6
	1.1 - 5	26	29.9
	5.1 or more	04	4.5
Religion	Catholicism	64	62.1
	Protestantism	30	29.2
	Spiritism	02	1.9
	Does not have	07	6.8
Practicing	Yes	61	59.2
	No	42	40.8

*Value of the minimum wage in effect at that time: R$ 678.00 (Decree 7,872, of December 26, 2012); †16 interviewees who refused to answer the question of family income.

As for the main causes of CKD, there was the prevalence of systemic arterial hypertension (53.4%), followed by type 2 diabetes mellitus (16.5%). Most interviewees (76.7%) reported willingness to undergo kidney transplantation. Regarding the type of vascular access, most respondents (95.2%) had arteriovenous fistula and some had (4.8%) double-lumen catheter (Table 2).

**Table 2:** Description of the clinical features of patients with chronic kidney disease. São Carlos, SP, Brazil, 2015.

Variable	Distribution by category	N	%
Baseline disease	Systemic arterial hypertension	55	53.4
	Diabetes	17	16.5
	Glomerulopathies	16	15.5
	Other Diseases	15	14.5
Type of access	Arteriovenous fistula	98	95.2
	Double-Lumen Catheter	05	4.8
Willingness to undergo transplantation	Yes	79	76.7
	No	24	23.3

Table 3 shows the results of social support perceived by patients with chronic kidney disease on hemodialysis. The average score of the instrumental social support was 3.81 (± 0.69), indicating a good availability of perceived support, considering that the score ranges from 1 to 5, and the higher the value, the better the social support. It is worth mentioning that 45.6% of participants said they were satisfied about the availability of support in the management and resolution of operational issues of treatment or health care, practical activities of daily living and material and/or financial assistance. 

**Table 3:** Distribution of factors of the Social Support Scale applied to patients on hemodialysis. São Carlos, SP, Brazil, 2014

Factor	Mean	Standard Deviation	From whom the support comes from	%
Instrumental Social support	3.81	0.69	Spouse, partner or boyfriend (girlfriend)	39.80
			Family person (s) living with me	55.35
			Family person (s) not living with me	34.95
			Friend (s)	9.72
			Neighbor (s)	0.98
			Other (s) person (s)	0.98
Emotional Social Support	3.92	0.78	Spouse, partner or boyfriend (girlfriend)	28.15
			Family person (s) living with me	38.83
			Family person (s) not living with me	34.95
			Friend (s)	37.86
			Boss or co-workers	0.97
			Neighbor (s)	1.94
			Health professionals	2.91
			Other (s) person (s)	3.89

With regard to the internal consistency, Cronbach's alpha was 0.76, indicating satisfactory reliability, and understanding and variability are likely to be interpreted.

As for the emotional social support, the average score was 3.92 (± 0.78), indicating a good satisfaction with the evaluated support. Is worth mentioning that many (50.6%) participants reported they were satisfied regarding the availability of listening, attention, information, esteem, companionship and emotional support (Table 3). Internal consistency was 0.90, indicating excellent reliability.

Among the most common sources of instrumental and emotional social support mentioned by the participants are the companions (spouse, partner or boyfriend), friends, family members - represented especially by the mother figure - sons and brothers were also cited (people living or not with the patient), health professionals. In addition, in the category others, interviewees mentioned the social support derived from religious institutions (Table 3).

## Discussion

The sample can be described in terms of the observed sociodemographic characteristics as composed predominantly of males. In addition, there were socioeconomic aspects such as having completed elementary school and the high prevalence of professionally inactive individuals, along with the incidence of hypertension as the main baseline disease for CKD^3^ ^,^ ^16^ ^-^ ^19^. 

In the search of studies on social support to patients with chronic kidney disease, it was observed a lack of research using the Social Support Scale as the data collection instrument. The findings were comparable to those observed for HIV and Heart Failure, which showed similar results to this study, regarding the satisfaction with the emotional social support^16^ ^,^ ^20^. 

The social support of patients with chronic kidney disease on hemodialysis, assessed by means of the Social Support Scale, had an average score of 3.92 for the emotional social support and 3.81 for the instrumental support. These results are very similar to those in the study, whose objective was to evaluate the social support of 85 outpatients with heart failure in the state of São Paulo, and the average score for the emotional social support was 3.8 and the average score for the instrumental support was 3.9. This shows that, although in different ways, patients with heart failure and CKD have a high level of social support, which is very important for the continuity of care^16^.

Social networks are a factor of health protection in terms of treatment and quality of life of HIV-positive people on treatment for HIV/AIDS. Studies have shown a correlation between social support, quality of life and adherence to treatment. Psychological aspects such as anxiety, depression and the perceived stress have an inverse correlation with the improvement in the health of patients living with HIV/AIDS^20^.

With regard to the patients with chronic kidney disease, the findings in the literature indicate that a good social support can increase the satisfaction of patients on dialysis with the care and quality of life related to health in general. In addition, social support can provide the means for a better treatment, adherence to medication and nutrition, leading to better clinical outcomes. Among the modalities of dialysis, the levels of social support and the associations may differ, as well as hemodialysis and peritoneal dialysis differ dramatically in terms of self-care required^10^ ^-^ ^11^.

Lower levels of social support were associated with increased risk of mortality and reduced adherence to treatment, particularly in relation to the duration of the dialysis session and weight gain, especially as regards the physical aspects^21^.

There is a significant association between the level of perceived social support and aspects of quality of life, such as the health functioning, socioeconomic aspects, spirituality and family relationships. Data shows the importance of considering the perception of social support when it comes to quality of life in patients with chronic kidney failure on hemodialysis^12^.

In Brazil, little attention has been given to the effects of a strong or weak social support network in the lives of patients on hemodialysis. Studies evaluating this variable are scarce and often use methods that do not allow generalization of results. Examples include studies^21^ ^-^ ^22^ using the qualitative approach. Although the data from these studies can not be generalized to the entire population of patients with chronic kidney disease, the results emphasize the importance of social support in the treatment and in the process of coping with the disease.

The limitation of this study is that patients evaluated belonged to a single unit of Renal Replacement Therapy in a municipality in the state of Sao Paulo. The results can not be generalized, since they show specific characteristics of a particular region of the country. However, these results provide an overview of the emotional and instrumental social support to patients with CKD on hemodialysis and can also provide useful information to support planning of care for these patients. 

Based on the proposed objectives and the results, it is concluded that, regarding the sociodemographic, economic and clinical characteristics found in this study, the findings corroborate the results of publications at national and international level with populations of patients with chronic kidney disease given that most respondents were male, self-declared white, Catholics, with low education and low income. 

Regarding the perceived social support, both instrumental support and emotional support, most CKD patients showed a satisfactory perception. It is worth mentioning that, based on the responses of the assessment instrument of perceived social support, specifically on the emotional social support; most interviewees reported they were satisfied regarding the availability of listening, attention, information, esteem, companion and emotional support. In relation to the instrumental social support, most interviewees reported they were satisfied with the availability of support in the management and resolution of operational issues of treatment or health care, practical activities of daily living and material and/or financial assistance. With respect to who offers this social support, both for the emotional and instrumental social support, most subjects reported receiving it from the family person (s) living with them.

Given that social support has been a factor that facilitates the coping with the disease and recovery of renal patients, it is suggest the inclusion of the assessment of perceived social support in the care planning. The assessment of such support could contribute to the detection of those individuals who have increased difficulty of medical rehabilitation, since little or no help can cause feelings of inability to change and maintain behaviors conducive to health.
